# Population Pharmacokinetics of Meropenem in Critically Ill Korean Patients and Effects of Extracorporeal Membrane Oxygenation

**DOI:** 10.3390/pharmaceutics13111861

**Published:** 2021-11-04

**Authors:** Dong-Hwan Lee, Hyoung-Soo Kim, Sunghoon Park, Hwan-il Kim, Sun-Hee Lee, Yong-Kyun Kim

**Affiliations:** 1 Department of Clinical Pharmacology, Hallym University Sacred Heart Hospital, Hallym University College of Medicine, Anyang 14066, Korea; dhlee97@hallym.or.kr; 2 Department of Thoracic and Cardiovascular Surgery, Hallym University Sacred Heart Hospital, Hallym University College of Medicine, Anyang 14066, Korea; cskhs99@hallym.or.kr (H.-S.K.); shlee1425@hallym.or.kr (S.-H.L.); 3 Division of Pulmonary, Allergy and Critical Care Medicine, Department of Internal Medicine, Hallym University Sacred Heart Hospital, Hallym University College of Medicine, Anyang 14066, Korea; f2000tj@hallym.or.kr (S.P.); hwanil@hallym.or.kr (H.-i.K.); 4 Division of Infectious Diseases, Department of Internal Medicine, Hallym University Sacred Heart Hospital, Hallym University College of Medicine, Anyang 14066, Korea

**Keywords:** meropenem, population pharmacokinetics, critically ill patient, adult, extracorporeal membrane oxygenation, Monte Carlo simulation

## Abstract

Limited studies have investigated population pharmacokinetic (PK) models and optimal dosage regimens of meropenem for critically ill adult patients using the probability of target attainment, including patients receiving extracorporeal membrane oxygenation (ECMO). A population PK analysis was conducted using non-linear mixed-effect modeling. Monte Carlo simulation was used to determine for how long the free drug concentration was above the minimum inhibitory concentration (MIC) at steady state conditions in patients with various degrees of renal function. Meropenem PK in critically ill patients was described using a two-compartment model, in which glomerular filtration rate was identified as a covariate for clearance. ECMO did not affect meropenem PK. The simulation results showed that the current meropenem dosing regimen would be sufficient for attaining 40%*f*T_>MIC_ for *Pseudomonas aeruginosa* at MIC ≤ 4 mg/L. Prolonged infusion over 3 h or a high-dosage regimen of 2 g/8 h was needed for MIC > 2 mg/L or in patients with augmented renal clearance, for a target of 100%*f*T_>MIC_ or 100%*f*T_>4XMIC_. Our study suggests that clinicians should consider prolonged infusion or a high-dosage regimen of meropenem, particularly when treating critically ill patients with augmented renal clearance or those infected with pathogens with decreased in vitro susceptibility, regardless of ECMO support.

## 1. Introduction

Antibiotic treatment is a major factor in determining the survival of critically ill patients diagnosed with sepsis. Altered pharmacokinetics (PK) in these patients is a major obstacle for clinicians when determining an adequate antibiotic dosage regimen [1]. The “third spacing” phenomenon caused by vasodilation and capillary leakage in sepsis patients increases the volume of distribution and lowers the drugs’ serum concentration, especially for hydrophilic antimicrobials [1,2], such as β-lactams and aminoglycoside, that are more affected by pathophysiological changes than lipophilic drugs [3].

Meropenem, a carbapenem β-lactam agent with a wide spectrum of activity against Gram-positive and Gram-negative pathogens, is used for the treatment of severe infections caused by multidrug-resistant organisms in intensive care unit (ICU) patients, including those on extracorporeal membrane oxygenation (ECMO) support [4,5,6,7]. A large heterogeneity was observed in the volume of distribution (over two-fold) in sepsis patients admitted to the ICU and receiving meropenem [3]. ECMO may further complicate PK changes in volume of distribution and clearance [8,9], which highlights the need for optimizing meropenem dosage in adult patients on ECMO. However, the effects of ECMO on the optimal dosage for meropenem have not been elucidated. Limited studies have performed population PK modelling and evaluated the pharmacodynamic (PD) alterations associated with ECMO [10,11,12]. Moreover, knowledge regarding the PK/PD profile of meropenem during ECMO and its clinical relevance for different ethnicities is also limited.

This study aimed to construct a population PK model for meropenem in critically ill Korean adult patients, including those receiving ECMO, to explore the effects of ECMO on meropenem PK. Moreover, we investigated optimal dosage regimens of meropenem by assessing the probability of PK/PD target attainment for various regimens using Monte Carlo simulations.

## 2. Methods

### 2.1. Patients

This prospective study was conducted in Hallym University Sacred Heart Hospital (840-bed university-affiliated tertiary referral hospital), Anyang, South Korea, from September 2020 to April 2021. Clinical indications for meropenem included: empirical management of sepsis from unknown source, nosocomial infections, and prophylactic administration for patients undergoing ECMO. Patients with a history of β-lactam allergy or a positive skin test result for meropenem were excluded. The demographic factors between the ECMO and the non-ECMO groups were compared. If meeting normality, the *t*-test was performed; otherwise, the Wilcoxon rank-sum test was used.

### 2.2. ECMO Apparatus

The ECMO system used was the Permanent Life Support (PLS) System (MAQUET, Rastatt, Germany), which consists of a broad range of HLS Cannulae and the Rotaflow Console. The PLS Set includes the PLS-i Oxygenator. A total of 1 L of plasma solution or normal saline was infused into the circuit, and the total circuit volume was 500–600 mL.

### 2.3. Study Design

Patients were able to participate at any time after the initiation of meropenem administration. Patients received 500 or 1000 mg of meropenem for 30 min every 8 or 12 h via intravenous (IV) infusion. Five blood samples were collected after the first dose following patients’ enrollment, and two samples were collected at steady state after four or five consecutive doses. The planned sampling times for model development were as follows: (1) immediately before dosing, (2) 0.5, 1, 4, and 8 h after the start of the infusion for the 8 h interval, and (3) 0, 0.5, 1, 6, and 12 h after the start of the infusion for the 12 h interval. Samples were collected before (trough level) and 30 min after (peak level) the fourth or fifth dosage, for model validation.

### 2.4. Meropenem Assay

Meropenem plasma concentrations were analyzed using high-performance liquid chromatography (HPLC)–tandem mass spectrometry (MS). The HPLC system consisted of a prominence LC-20A System (Shimadzu, Japan) and a Gemini C_18_ column (Kinetex; Phenomenex, Torrance, CA, USA). The MS detection was conducted using a hybrid triple quadrupole/Linear Ion trap mass spectrometer (API4000 QTRAP; SCIEX, Framingham, MA, USA). Briefly, a 100 μL aliquot of plasma sample was pipetted into a centrifuge tube. Next, 100 μL of acetonitrile containing an internal standard (20 μg/mL, ceftazidime) was added to the tube and then vortexed for 1 min. After centrifugation at 12,000 rpm for 2 min, the supernatant was transferred to another centrifuge tube, and 100 μL of 0.1% formic acid was added to the tube. An aliquot of 10 μL was injected into the LC–MS/MS system. The lower limit of quantitation was 0.2 mg/L. The assay results were linear over a range from 0.2 to 200 mg/L (R^2^ = 0.99). Intraday precision and accuracy of the validation concentration range (0.5, 5, and 50 mg/L) analyzed using standard samples were 2.98–3.92% and 96.53–110.07%, respectively. Interday precision and accuracy of the validation concentration range (0.5, 5, and 50 mg/L) analyzed using standard samples for 3 days were 0.5–2.7% and 89.9–100.0%, respectively.

### 2.5. Population PK Analysis

Population PK modeling was implemented using the Nonlinear-mixed effects modelling software (NONMEM^®^ 7.5, ICON Development Solutions, Elliot City, MD, USA). The first-order conditional estimation with interaction (FOCEI) method was used to estimate measured (fixed) and unexplained (random) effect parameters. FOCEI allows the interaction between the inter-individual variability (IIV, η) of PK parameters and the residual variability (RV) of measured concentrations. RV was caused by inter-individual variability, measurement error, assay error, and model misspecification. One-, two-, and three-compartment structural models were investigated using the PK model library in NONMEM. All PK processes were assumed to follow first-order kinetics rather than zero-order infusion. The PK parameter was defined as θ_i_ = θ × exp(η_i_), where θ is the typical value of the PK parameter, θ_i_ is an individual PK parameter, and η_i_ is a random effect associated with IIV, which is assumed to have a normal distribution with a mean of 0 and a variance of ω^2^. Proportional, additive, or combined proportional and additive error models were tested for RV, which was assumed to have a normal distribution with a mean of 0 and a variance of σ^2^. A power parameter was tested to allow for nonlinear heteroscedastic variances [13].

Models were evaluated and selected based on NONMEM objective function values (OFVs), precision of parameter estimates (relative standard errors), shrinkage of IIV, and diagnostic goodness-of-fit plots. In a log-likelihood ratio test, a decrease in the OFV (ΔOFV) between two nested models, having 1 degree of freedom greater than 3.84 or 2 degrees of freedom greater than 5.99, was considered statistically significant at *p* < 0.05 for model improvement. Diagnostic plots included the following four plots: conditional weighted residuals (CWRES) vs. time, CWRES vs. model-predicted population concentration (PRED), observation vs. PRED, and observation vs. model-predicted individual concentration.

Perl-speaks-NONMEM software (PSN, version 5.2.6, available online: https://uupharmacometrics.github.io/PsN, accessed on 17 June 2021)) was used for searching covariates, evaluating a model with visual predictive check and conducting nonparametric bootstrap to obtain 95% confidence intervals (CIs). To search significant covariates for the PK parameters, stepwise forward inclusion and backward exclusion processes were conducted. Statistical significance was set at *p* < 0.01 (ΔOFV < −6.635 for 1 degree of freedom) for inclusion and *p* < 0.001 (ΔOFV > 10.83 for 1 degree of freedom) for exclusion. A significant covariate should have both statistical significance and clinical relevance. The tested covariates for structural PK parameters were age, sex, height, weight, body surface area (BSA), serum albumin level, serum protein level, serum creatinine level, serum cystatin C level, primary diagnosis, comorbidity, renal function, ECMO type (veno–arterial (VA) or veno–venous (VV)), and ECMO flow rate. The renal function was calculated by applying Chronic Kidney Disease Epidemiology Collaboration (CKD-EPI), modified CKD-EPI, Modification of Diet in Renal Disease (MDRD), modified MDRD, and Cockcroft-Gault (CG) formulations to determine the total clearance (CL). The modified CKD-EPI and MDRD estimates were adjusted using individual BSA values, where BSA was calculated by applying the Du Bois formula. Visual predictive check with prediction and variability correction (VPC_PVC_) was performed using PSN by comparing the final PK model with the measured plasma concentrations with 80% prediction intervals from 1000 virtual datasets. Nonparametric bootstrapping was performed to investigate the stability of the final PK model. The median and 95% confidence interval for the estimates of bootstrap samples (*n* = 2000) were generated to evaluate the parameter estimates of the final PK model. R software (version 4.0.4, available online: www.rproject.org, accessed on 11 March 2021) was used for the postprocessing of model output and visualization. 

The individual PK parameters between ECMO and non-ECMO groups were compared. If meeting normality, an independent *t*-test was used; otherwise, Wilcoxon rank-sum test was used.

### 2.6. Assessment of Prediction Performance

The predictive performance of the final PK model was assessed visually using the relative prediction error (rPE) vs. the observed concentration plot and numerically using the relative bias (rBias) for accuracy and the relative root-mean-square error (rRMSE) for precision.

$$\mathrm{rPE}=\frac{C_{P}-{\;C}_{O}}{C_{O}}$$

$$\mathrm{rBias}=100\%\frac{1}{N}\underset{i}{\sum }\frac{C_{P}-{\;C}_{O}}{C_{O}}$$

$$\mathrm{rRMSE}=100\%\sqrt{\frac{1}{N}\underset{i}{\sum }\frac{{\left(C_{P}-{\;C}_{O}\right)}^{2}}{C_{O}^{2}}}$$

where C_O_ indicates the observed concentrations, and C_P_ v the predicted concentrations. 

### 2.7. PD Target Attainment

Four Monte Carlo simulations were implemented. The first simulation was conducted to explore the adequacy of the recommended dosage regimen (for a creatinine clearance [CL_CR_] > 50 mL/min, 1 g every 8 h by i.v. infusion; for a CL_CR_ of 26–50 mL/min, 1 g every 12 h by i.v. infusion; for a CL_CR_ of 10–25 mL/min, 500 mg every 12 h by i.v. infusion; for a CL_CR_ < 10 mL/min, 500 mg every 24 h i.v. infusion) when treating adult patients infected with *Pseudomonas aeruginosa* (*P. aeruginosa*). A total of 10,000 individual PK parameters were generated for virtual patients assuming a log-normal distribution for each parameter with the typical parameter values and the IIV of the final PK model. The selected covariate, glomerular filtration rate (eGFR) estimated using the CKD-EPI equation, was generated assuming a log-normal distribution within the range of 0 to 130 mL/min. Patients were treated empirically without knowing the pathogen, while minimum inhibitory concentration (MIC) values were generated using the clinical breakpoint distribution of MICs set by the European Committee on Antimicrobial Susceptibility Testing. They were randomly assigned to the 10,000 virtual patients. The steady-state concentration–time profiles of the virtual patients were generated using the simulated individual PK parameters and the recommended dosage regimen.

The antimicrobial activity of meropenem is related to the cumulative percentage of a 24 h period during which the free drug (unbound to protein, *f*) concentration exceeds the MIC for a pathogen, in steady-state condition (*f*T_>MIC_). The parameter *f* was fixed at 98%. The tested treatment targets were 40%*f*T_>MIC_, 100%*f*T_>MIC_, and 100%*f*T_>4XMIC_. A dosage strategy was considered adequate if the probability of target attainment (PTA) was greater than or equal to 90%. The PTAs for treatment target were compared for various combinations of renal function, MICs, and dosage regimen of the patients.

The second, third, and fourth simulations were conducted to determine the optimal dosage regimen for 40%*f*T_>MIC_, 100%*f*T_>MIC_, and 100%*f*T_>4XMIC_ as treatment targets, respectively. A total of 1000 individual PK parameters were generated for virtual patients assuming a log-normal distribution for each parameter, whereas the covariate was generated by applying a uniform distribution within the range 0 to 170 mL/min. The patients were divided into the six renal function groups (0 < CL_CR_ ≤ 10, 10 < CL_CR_ ≤ 25, 25 < CL_CR_ ≤ 50, 50 < CL_CR_ ≤ 90, 90 < CL_CR_ ≤ 130, and 130 < CL_CR_ ≤ 170 mL/min). Steady-state concentration–time profiles of the 1000 virtual patients were generated for various combinations of the three doses (0.5, 1, and 2 g), two dosing intervals (8 and 12 h), four infusion times (0.5, 1, 2, and 3 h), and MICs (0.060, 0.125, 0.25, 0.5, 1, 2, 4, 8, and 16 mg/L). 

## 3. Results

### 3.1. Patient Characteristics 

The demographic and clinical characteristics of the 26 patients are described in Table 1. Eight adult patients received ECMO (veno–arterial (VA) ECMO, *n* = 7; veno–venous (VV) ECMO, *n* = 1). One of the 18 patients in the non-ECMO group and one of the eight patients in the ECMO group received continuous renal replacement therapy. Patients on ECMO support were younger (median age, interquartile range [IQR]; 64.0 [56.3–66.5] vs. 72.0 [66.0–80.3] days; *p* = 0.0167). The severity scores, including the APACHE II (median [IQR]; 12 [10,11,12,13,14] vs. 10 [7,8,9,10,11,12,13,14]; *p* = 0.0321) and SOFA scores (median [IQR]; 9.50 [8.00–12.5] vs. 5.00 [3.00–7.75]; *p* = 0.0051), were significantly higher in patients in the ECMO group compared to those in the non-ECMO group. 

### 3.2. Population PK Analysis

A total of 125 samples were used to develop a population PK model, and 44 samples to validate the final model. The concentration–time profile of meropenem was best described by a two-compartment model. The NONMEM OFVs for one-, two-, and three-compartment models were 689.840, 6540.693, and 640.694, respectively.

The structural parameters for the two-compartment model were total clearance (CL), central volume of distribution (V_C_), peripheral volume of distribution (V_P_), and intercompartmental clearance (Q) between V_C_ and V_P_. The inter-individual variability (IIV) was estimated for CL, V1, and V2 (Table 2). In the final PK model (OFV 611.402), GFR was estimated using the CKD-EPI equation and identified as a statistically significant covariate of CL. The IIV for CL was reduced from 55.2% to 31.4% after the covariate were included. ECMO therapy did not affect the meropenem PK in this study.

The PK parameter estimates were not significantly different between the ECMO and the non-ECMO groups (Table 3). Residual error was well described using a proportional error model. The power parameter for RV reduced the relative standard error (RSE) of the IIV for CL from 27.3% to 22.5%. When IIV was expressed as a standard deviation, if the RSE of IIV exceeded 25%, it was not significant.

**Figure 1:**
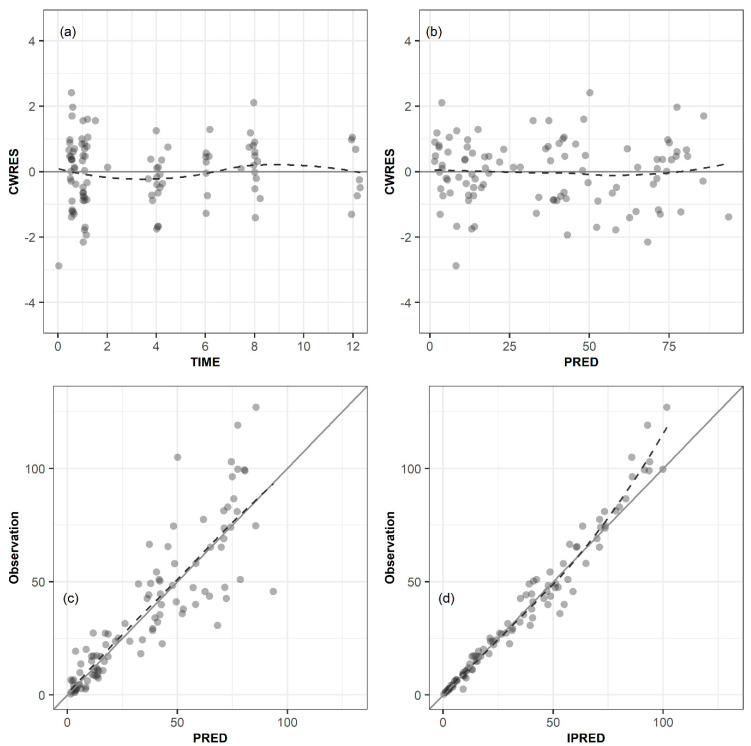
Goodness–of–fit plots. (**a**) Conditional weighted residuals (CWRES) versus time, (**b**) CWRES versus population predicted concentration (PRED), (**c**) observed concentration versus PRED, and (**d**) observed concentration versus individual predicted concentration. The dashed lines indicate smooth curves.

The diagnostic goodness-of-fit plots for the final PK model are depicted in Figure 1. Conditional weighted residuals (CWRES) were randomly distributed around the x-axis, indicating no systemic deviation in the structural model (Figure 1a) or in the residual error model (Figure 1b); most of them remained within ±2 times the normalized standard deviation. The observation values were randomly distributed around the line of identity, indicating no evidence of misspecification of the structural, IIV, or RV model (Figure 1c,d). 

**Figure 2:**
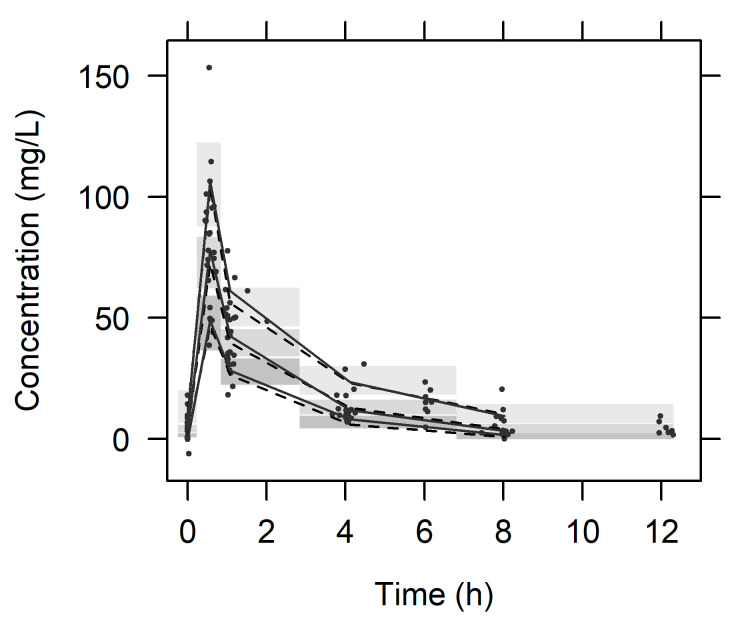
Visual predictive check plot. Plots from virtual concentrations of 1000 simulated datasets. Closed circles, observed serum meropenem concentrations; solid lines, the 10th, 50th, and 90th percentiles of observations; dashed lines, the 10th, 50th, and 90th percentiles of simulated serum meropenem concentrations; shaded areas, 95% confidence intervals for each percentiles of simulated concentrations.

VPC_PVC_ is shown in Figure 2, where most of the observations fall within the 80% prediction interval of the simulated concentrations, and the observed 10th, 50th, and 90th percentiles are overlaid with 95% CIs of the simulated 10th, 50th, and 90th percentiles. This plot suggests that the final PK model correctly explained the data and had appropriate predictive performance. The time course for individual observed, individual predicted, and population predicted concentrations is shown in Figure S1.

### 3.3. Assessment of Prediction Performance

Figure S2 displays the relative prediction error (rPE) vs. the observed concentration. As shown, most values are distributed around the x-axis. When the concentration exceeded 50 mg/L, underprediction was observed. When all subjects were included, the relative bias (rBias) and relative root-mean-square error (rRMSE) were 17.5% and 91.5%, respectively. However, when two subjects with extreme outliers were excluded, the rBias and rRMSE were 1.59% and 29.4%, respectively. 

### 3.4. PD Target Attainment

**Figure 3:**
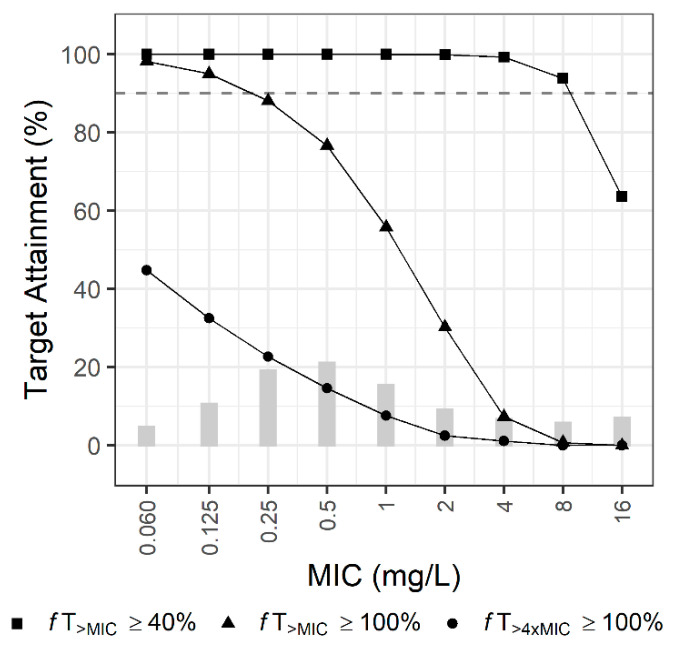
Probabilities of target attainment (PTA) of empirical therapy using the current dosage regimen for patients with an eGFR of 0–130 mL/min/1.73 m^2^. Bars indicate the MIC distribution for *P. aeruginosa*.

Figure 3 shows the PTA of empirical therapy using the current dosage regimen in the first simulation. The recommended dosage regimen achieved 90% PTA at 40%*f*T_>MIC_ when the MIC was less than 8 mg/L; however, it did not achieve 90% PTA at 100%*f*T_>MIC_ when the MIC was greater than 0.25 mg/L. If the target was 100%*f*T_>4XMIC_, the current regimen could not reach the 90% PTA regardless of the MIC.

**Figure 4:**
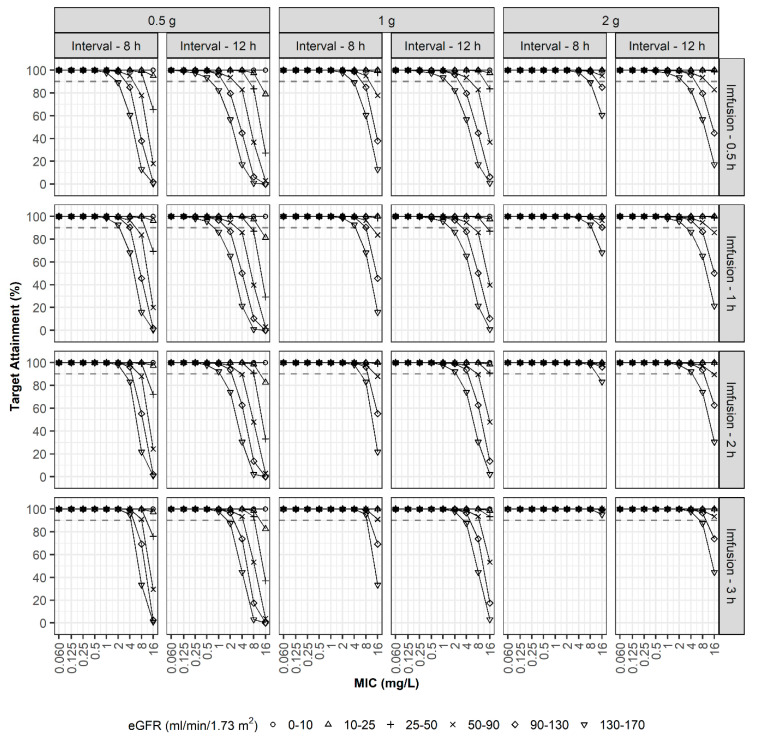
PTAs (40%*f*T_>MIC_). Simulation results with three doses (0.5, 1, and 2 g), two dosing intervals (8 and 12 h), four infusion times (0.5, 1, 2, and 3 h), various degrees of renal function, and various MICs.

In the second simulation, optimal dosage regimens were explored to achieve PTA > 90% at 40%*f*T_>MIC_ (Figure 4). In the case of patients with an eGFR of 26–50 mL/min/1.73 m^2^, a regimen of 1 g every 12 h using i.v. infusion over 30 min could attain a 90% PTA when the MIC was 4 mg/L. However, a dosage regimen of 0.5 g every 12 h was also appropriate for patients in this study. As expected, a prolonged infusion enhanced the PTA. For patients with eGFR values of 90–130 mL/min/1.73 m^2^, a dosage regimen of 1 g every 12 h using i.v. infusion over 2 h was optimum when the MIC was 4 mg/L, whereas 30 min or 1 h of infusion was not.

**Figure 5:**
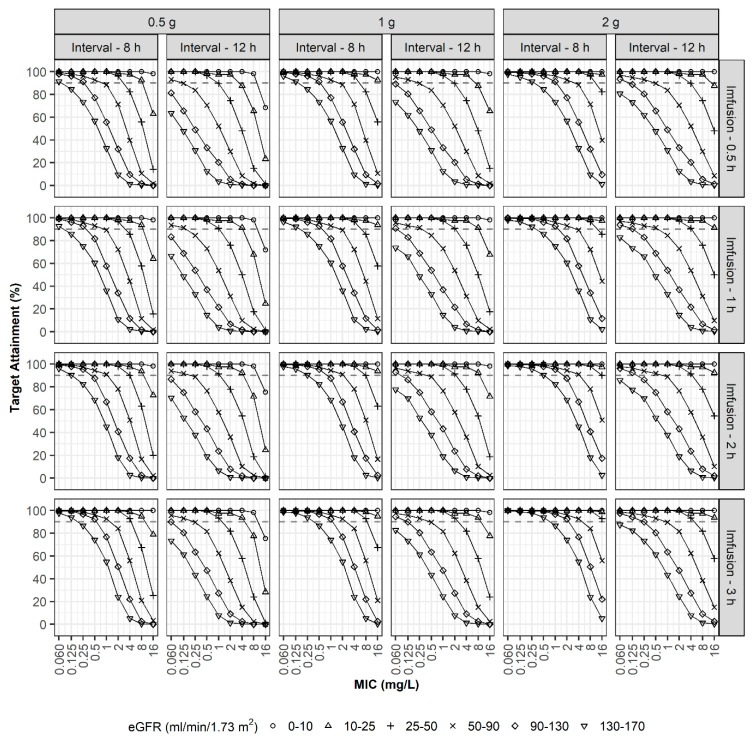
PTAs (100%*f*T_>MIC_). Simulation results with three doses (0.5, 1, and 2 g), two dosing intervals (8 and 12 h), four infusion times (0.5, 1, 2, and 3 h), various degrees of renal function, and various MICs.

In the third simulation, optimal dosage regimens were explored to achieve PTA > 90% at 100%*f*T_>MIC_ (Figure 5). In the case of patients with an eGFR of 50–90 mL/min/1.73 m^2^, a regimen of 1 g every 8 h using i.v. infusion over 30 min or prolonged infusion over 3 h could attain a 90% PTA when the MIC was 1 mg/L and 2 mg/L, respectively. For patients with augmented renal clearance (eGFR values of 130–170 mL/min/1.73 m^2^), a regimen of 1 g every 8 h using prolonged i.v. infusion over 3 h was optimum only when the MIC was equal to or less than 0.25 mg/L, and a high-dosage regimen of 2 g every 8 h using prolonged i.v. infusion over 3 h was optimum when the MIC was less than 1 mg/L.

**Figure 6:**
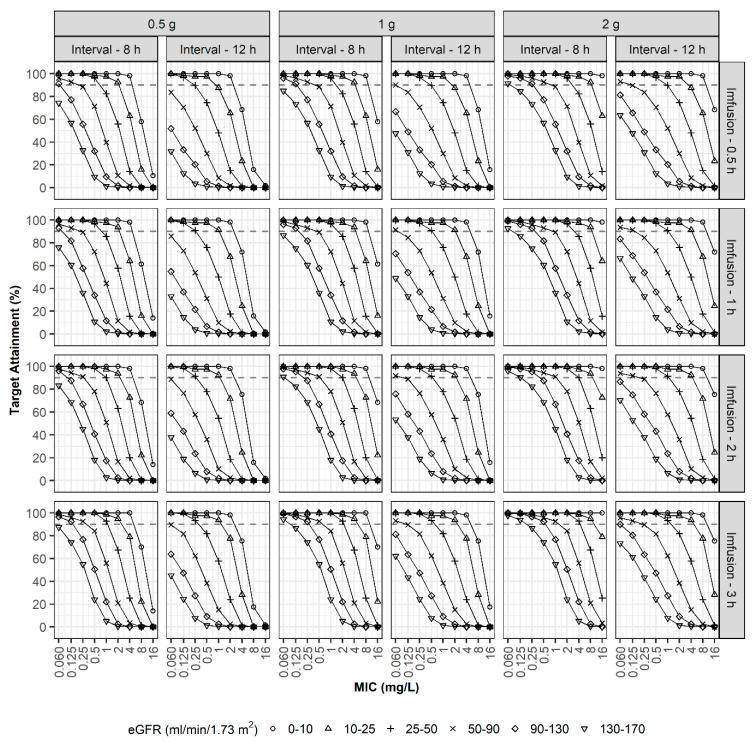
PTAs (100%*f*T_>4XMIC_). Simulation results with three doses (0.5, 1, and 2 g), two dosing intervals (8 and 12 h), four infusion times (0.5, 1, 2, and 3 h), various degrees of renal function, and various MICs.

In the fourth simulation, optimal dosage regimens were explored to achieve PTA > 90% at 100%*f*T_>4XMIC_ (Figure 6). In the case of patients with an eGFR of 50–90 mL/min/1.73 m^2^, a regimen of 1 g every 8 h using i.v. infusion over 30 min could attain a 90% PTA when the MIC was ≤0.25 mg/L, and the high-dosage regimen of 2 g every 8 h using prolonged i.v. infusion over 3 h could attain a 90% PTA when the MIC was ≤1 mg/L. For patients with augmented renal clearance (eGFR values of 130–170 mL/min/1.73 m^2^), the high-dosage regimen of 2 g every 8 h using prolonged i.v. infusion over 3 h was optimum only when the MIC was less than 0.25 mg/L.

## 4. Discussion

This study presents the PK properties of meropenem in critically ill Korean adult patients, including those undergoing ECMO. The PK of meropenem was best described by a two-compartment model, in which the glomerular filtration rate (GFR) was estimated using the CKD-EPI equation and identified as a significant covariate for CL. The final PK model demonstrated good predictive performance, while using ECMO did not affect meropenem PK. The results of Monte Carlo simulations to achieve more than 90% PTA at 40%*f*T_>MIC_ suggest that the current dosage regimen of meropenem using i.v. infusion over 30 min is sufficient to treat *P. aeruginosa* at MIC ≤ 4 mg/L in the case of patients with a CL_CR_ of 50–90 mL/min/1.73 m^2^. Prolonged i.v. infusion of meropenem over at least 2 h could attain a 90% PTA when the MIC is 4 mg/L for patients with augmented renal clearance (eGFR values of 130–170 mL/min/1.73 m^2^). However, to achieve PTA > 90% at 100%*f*T_>MIC_ or 100%*f*T_>4XMIC_, prolonged infusion over 3 h or a high-dosage regimen of 2 g every 8 h should be considered, particularly for MIC > 2 mg/L or in patients with an eGFR of 130–170 mL/min/1.73 m^2^.

Prescribing an antibiotic with a narrow therapeutic range is one of the most challenging concerns in the treatment of critically ill patients with time-varying and highly variable PK. The current paucity of knowledge about PK regarding ECMO use in critically ill patients makes it more difficult to adjust dosage regimens. Regardless of ECMO support, PTA for achieving 100%*f*T_>MIC_ and 100%*f*T_>4XMIC_ in the present study was low when meropenem was used following the currently recommended dosage regimen to treat even susceptible *P. aeruginosa* (MIC ≤ 2 mg/L) in patients with normal renal clearance. These results are consistent with those of previous studies that performed population PK model-based simulations in critically ill patients, including those on ECMO support [10,11,12]. We postulate that prolonged infusion of meropenem over 3 h or a high-dosage regimen of 6 g/day should be considered, particularly for treating critically ill patients with increased renal clearance and those infected with less susceptible pathogens, to provide appropriate meropenem concentrations to them and those on ECMO support. Real-time therapeutic drug monitoring (TDM)-guided dosing optimization of meropenem using dosing software could further help clinicians to improve clinical outcomes and reduce toxicity in critically ill patients [14,15,16]. Evaluating the influence of TDM of meropenem on clinical outcomes in ECMO patients is warranted, since the use of inappropriate meropenem concentrations is possible, especially when using the standard-dose regimen [17]. 

Regarding poor clinical outcomes associated with elevated meropenem MIC [18,19,20], the development of guidance for appropriate meropenem dosage optimization for empirical treatment should be considered to overcome antimicrobial resistance in the ICU [21]. High-dosage regimens of meropenem using prolonged infusion over 3 h could be considered for the empirical treatment in critically ill patients [22,23,24,25], which is supported by our simulation results revealing the low PTA of empirical therapy using the current dosing regimen. Previous studies have reported the positive impact of prolonged meropenem infusion on clinical outcomes such as hospital mortality in critically ill patients [26,27]. Providing patients with ECMO support may help to better evaluate the influence of prolonged infusion or high-dosage regimen on clinical outcomes in critically ill patients.

The PK profile of meropenem in our study was well described by a two-compartment model. Model-predicted typical values of CL and steady-state volume of distribution (V_SS_ = V_C_ + V_P_) for meropenem were 6.37 L/h and 17.0 L, respectively. These values were consistent with the results of previous population PK studies including patients with mild to severe renal impairment (CL, 2.0–7.7 L/h; V_SS_ 14.2–26.7 L) [28,29] or patients on ECMO (CL, 2.79–14.7 L/h; V_SS_, 15.3–33.6 L) [10,11,12]. The mean (s.d.) CL and V_SS_ of individual estimates for patients without ECMO support were 5.90 (3.60) L/h and 17.8 (5.47) L, respectively, while those for patients on ECMO support were 6.55 (2.14) L/h and 17.0 (2.70) L, respectively. There were no differences between the two groups when independent *t*-tests for CL (*p* = 0.5782) and V_SS_ (*p* = 0.6140) were performed after the Shapiro–Wilk normality test, which was consistent with the results of three population PK studies involving ECMO patients that found no effect of ECMO on meropenem PK [10,11,12]. In these three studies, which included 10 to 14 patients on ECMO, the presence or absence of ECMO was not selected as a covariate affecting PK parameters, and ECMO factors such as VA/VV types, flow rate, or pump speed were not included as covariates in the pharmacokinetic model [10,11,12]. Shekar et al. compared the results of a population PK analysis of 11 ECMO patients with previously published data from 10 non-ECMO patients and found that ECMO patients demonstrated reduced meropenem CL and an increased V_SS_ when compared with controls, but these changes were not statistically significant [10]. Gijsen et al. compared 14 ECMO patients with 11 non-ECMO patients in one study and found no PK alteration by ECMO use [12]. We believe that variations in meropenem PK among critically ill patients of different races, regardless of ECMO support, are limited because the renal clearance of meropenem is largely dependent on the passive process of glomerular filtration [30,31,32].

We tested the effect of renal function on meropenem clearance. When renal function was estimated using Cockcroft–Gault (CG), Modification of Diet in Renal Disease (MDRD), modified MDRD, CKD-EPI, and CKD-EPI equations on the basic PK model, the OFV decreased by 24.512, 24.948, 22.202, 27.933, and 24.270, respectively. Therefore, the eGFR estimated using the CKD-EPI equation was considered the best covariate explaining the individual variation in CL. In previous population PK studies of meropenem, renal function calculated by CG equation [10,11,28,33,34,35], CKD-EPI formula [12], or MDRD [29,36] formula was selected as a covariate affecting CL. The difference in the reduction of OFV was not substantial in our study; there would have been no significant differences in the predictive performance of the model even if other formulas for renal function were used. In our study, only GFR estimated by the CKD-EPI equation was included as a covariate affecting CL, while a recently developed model included body weight and eGFR as covariates [12]. However, since the coefficients of body weight were fixed, only eGFR was the covariate selected by the modeling process.

The predictive performance of the final PK model was evaluated using an external validation dataset. Predictive errors of 203% and 564% were observed in two patients. The two samples were collected immediately prior to dosing but were recorded as being collected after initiating dosing; it was more likely to be an error in the documentation than an error in the conduct of the clinical trial. However, since the exact cause could not be identified, we analyzed all data and had poor predictive performance. When the two subjects were excluded, rBias and rRMSE decreased from 17.5% to 1.59% and from 91.5% to 29.4%, respectively, showing that our model has good predictive ability. The range of rBias and rRMSE of the tobramycin model that showed good performance ranged from 4.9% to 29.4% and from 47.8% to 66.9%, respectively [37].

Our study has some limitations. First, the number of patients on ECMO support (*n* = 8) was too small to accurately detect the effect of ECMO on meropenem PK parameters, although the total number of patients (*n* = 26) was sufficient to construct a population PK model. Given the physicochemical properties of meropenem, ECMO therapy may not significantly affect its PK. Second, only one covariate was included in the final PK model; hence, it is difficult to directly use this model for personalized treatments of patients. It is necessary to improve the model through follow-up studies. Third, the predictive performance of the final PK model for 26 patients was not good. The number of blood samples used for external validation was small and substantially affected by two extreme values. However, our model could accurately predict most meropenem concentrations for validation. Fourth, we did not validate our results of PK/PD analysis by evaluating clinical outcomes. Despite these limitations, our study is valuable because it is, to the best of our knowledge, the first population PK analysis of meropenem using model-based simulations in critically ill Korean patients, including those on ECMO support. We believe that the present study has important clinical implications for future research to develop the optimal dose recommendation for such important populations.

## 5. Conclusions

Our model shows that ECMO use does not affect meropenem PK in critically ill patients. Our simulation results suggest that the current dosing regimen of meropenem using i.v. infusion over 30 min may be related to a suboptimal concentration for empirical treatment in ICU. Therefore, prolonged i.v. infusion over 3 h or a high-dosage regimen of 2 g every 8 h should be considered, particularly at MIC > 2 mg/L or in patients with augmented renal clearance. Further studies are warranted to validate our model-based regimen, such as assessing the clinical outcomes and evaluating the influence of TDM on clinical outcomes in critically ill patients, including those on ECMO support.

## Figures and Tables

**Table 1:** Patients’ characteristics (median (IQR)).

Parameter	ECMO (*n* = 8)	Non-ECMO (*n* = 18)	*p*-Value
ECMO type	VA 7/VV 1		
CRRT	Yes 3/No 5	Yes 1/No 17	
Sex	male 4/female 4	male 14/female 4	
Age (year)	64.0 (56.3–66.5)	72.0 (66.0–80.3)	0.0167 ^c^
Height (cm)	162 (153–169)	165 (156–170)	0.6544 ^d^
Weight (kg)	63.5 (61.9–66.3)	54.4 (50.5–64.5)	0.1731 ^d^
Body surface area (m^2^)	1.67 (1.61–1.75)	1.63 (1.51–1.72)	0.5074 ^c^
ICU duration (days)	25.0 (6.00–43.5)	6.50 (4.00–17.8)	0.1249 ^d^
APACHE II	21.0 (19.5–22.5)	16.0 (12.0–18.0)	0.0321 ^c^
SOFA	9.50 (8.00–12.5)	5.00 (3.00–7.75)	0.0051^c^
BUN (mg/dL)	26.9 (22.0–33.0)	22.6 (10.8–46.6)	0.8675 ^d^
Scr (mg/dL)	0.820 (0.518–1.15)	0.615 (0.458–1.43)	0.9557 ^d^
Cystatin C (mg/dL)	1.48 (1.43–1.90)	1.34 (0.985–1.86)	0.5411 ^c^
Albumin (g/dL)	3.00 (2.83–3.20)	2.55 (2.30–2.98)	0.0364 ^c^
Protein (g/dL)	5.15 (4.88–5.75)	5.05 (4.70–5.75)	0.5448 ^d^
CL_CR_, Cockcroft-Gault (mL/min)	76.9 (59.5–105)	73.4 (32.7–92.6)	0.4367 ^d^
GFR, MDRD (mL/min/1.73 m^2^)	86.9 (67.1–132)	111 (47.5–160)	0.9119 ^c^
GFR, modified MDRD (mL/min) ^b^	88.7 (67.0–115)	95.1 (45.0–153)	0.8676 ^d^
GFR, CKD-EPI (mL/min/1.73 m^2^)	87.7 (70.0–105)	91.6 (45.6–103)	0.7145 ^c^
GFR, modified CKD-EPI (mL/min) ^b^	82.4 (70.0–94.9)	77.7 (43.6–97.0)	0.5883 ^c^

IQR, interquartile range; ECMO, extracorporeal membrane oxygenation; VA, veno–arterial; VV, veno–venous; CRRT, continuous renal replacement therapy; APACHE II, Acute Physiology and Chronic Health Evaluation; SOFA, sequential organ failure assessment; BUN, serum blood urea nitrogen level; Scr; serum creatinine level; CL_CR_, creatinine clearance; GFR, glomerular filtration rate; MDRD, Modification of Diet in Renal Disease; CKD-EPI, Chronic Kidney Disease Epidemiology Collaboration. ^b^ The modified MDRD and CKD-EPI equations adjusted to individual BSA are GFR (mL/min) = GFR (MDRD or CKD-EPI) × (BSA/1.73 m^2^). ^c^ Independent *t*-test. ^d^ Wilcoxon rank-sum test.

**Table 2:** Population PK parameter estimates for meropenem.

Parameter	Estimates	RSE (%) [Shrinkage (%)]	Bootstrap Median (95% CI)
Structural model			
CL = θ_1_ × (1 + θ_2_ × (CE − 91.57))			
θ_1_ (L/h)	6.37	7.41	6.32 (5.42–7.23)
θ_2_	0.00925	10.3	0.00932 (0.00680–0.0110)
V_C_ (L)	9.07	12.2	8.97 (3.92–12.0)
Q (L/h)	10.7	21.5	10.6 (4.73–31.0)
V_P_ (L)	7.91	13.6	8.17 (5.35–11.1)
Inter-individual variability			
CL (%)	31.4	15.8 [3.70]	29.9 (18.0–38.7)
V_C_ (%)	43.6	22.5 [14.7]	41.0 (0.000–95.4)
V_P_ (%)	36.6	21.0 [41.3]	34.5 (0.000–55.7)
Residual variability			
Proportional error (%)	24.6	29.3 [24.2]	24.1 (10.3–41.5)
Power parameter	0.865	10.0	0.897 (0.533–1.38)

ECMO, extracorporeal membrane oxygenation; RSE, relative standard error; CL, total clearance; θ_1_, typical population value for CL; θ_2_, covariate coefficient for CE; V_C_, central volume of distribution; V_P_, peripheral volume of distribution; Q, inter-compartmental clearance between V_C_ and V_P_; CE, glomerular filtration rate estimated by CKD-EPI equation.

**Table 3:** Comparison of population PK parameter estimates between the ECMO and the non ECMO groups.

Parameter	ECMO	non ECMO	*p*-Value
CL	6.34 (4.84–7.92)	5.05 (3.43–7.37)	0.5782 ^b^
V_C_ (L)	8.37 (7.35–8.89)	8.53 (7.21–11.7)	0.6567 ^c^
V_P_ (L)	8.11 (7.75–8.86)	8.28 (6.55–8.64)	0.4258 ^b^
V_SS_ (L)	16.2 (15.6–17.9)	17.2 (14.7–21.0)	0.6140 ^b^

ECMO, extracorporeal membrane oxygenation; CL, total clearance; V_C_, central volume of distribution; V_P_, peripheral volume of distribution; V_SS_, steady-state volume of distribution. ^b^ Independent *t*-test. ^c^ Wilcoxon rank-sum test.

## Data Availability

The datasets generated and/or analyzed during the current study are available from the corresponding author on reasonable request.

## Supplementary Materials

The following are available online at https://www.mdpi.com/article/10.3390/pharmaceutics13111861/s1, Figure S1: Individual fit plots. Closed circle, observed concentration; solid line, individual-predicted concentrations; dotted line, population-predicted concentrations, Figure S2: Relative prediction errors vs observations for the final population pharmacokinetic model of meropenem: (a) plots for all subjects (*n* = 26), (b) plots excluding the two patients with extreme values (*n* = 24).
