# Supplementary material for: Population Pharmacokinetics of Meropenem in Critically Ill Korean Patients and Effects of Extracorporeal Membrane Oxygenation
Source: Pharmaceutics. 2021 Nov 4;13(11):1861. doi: 10.3390/pharmaceutics13111861 (PMC8625191; doi:10.3390/pharmaceutics13111861)
Supplement: Supplementary file 1 [file pharmaceutics-13-01861-s001.zip › pharmaceutics-1422854-supplementary.pdf]

pharmaceutics

MDPI

Population Pharmacokinetics of Meropenem in Critically Ill Korean Patients and Effects of Extracorporeal Membrane Oxygenation

Dong-Hwan Lee , Hyoung Soo Kim , Sunghoon Park , Hwan-il Kim , Sun Hee Lee and Yong Kyun Kim

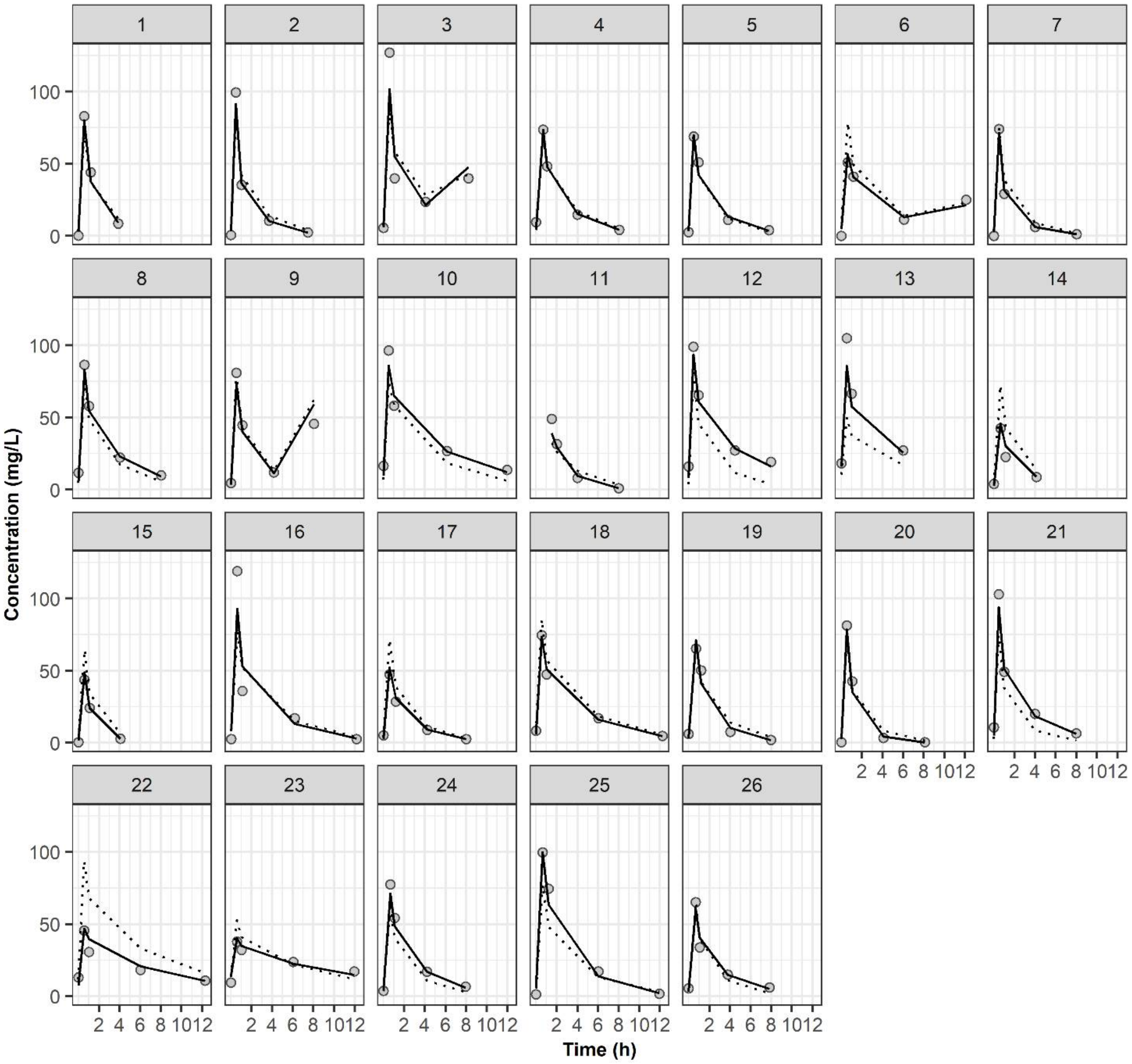

Figure S1. Individual fit plots. Closed circle, observed concentration; solid line, individual-predicted concentrations; dotted line, population-predicted concentrations.

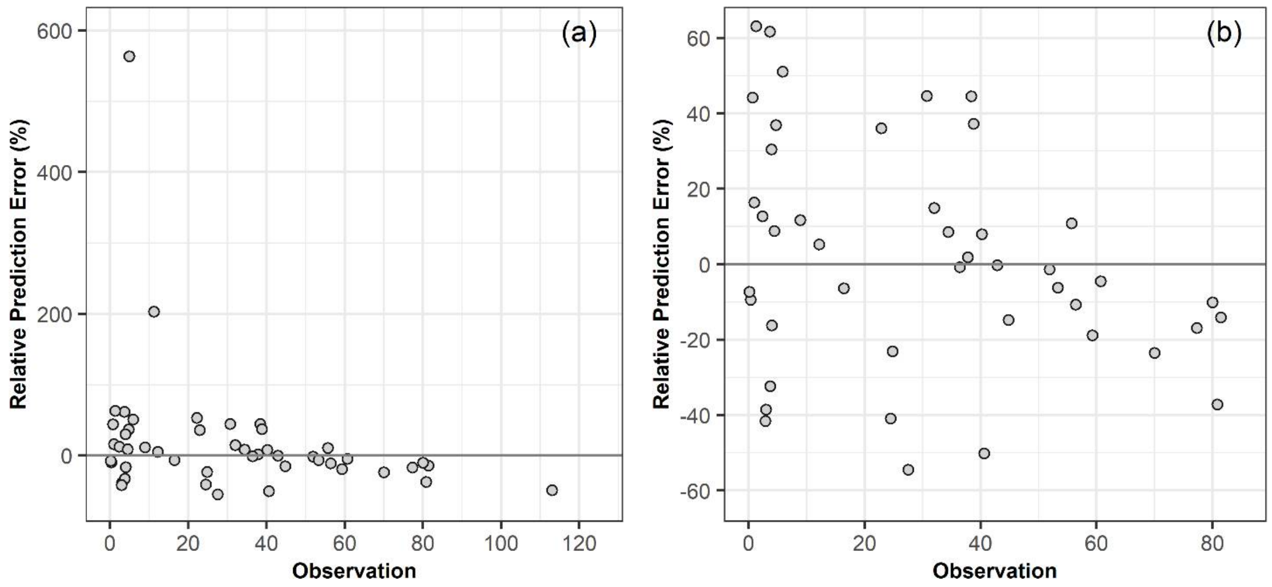

Figure S2. Relative prediction errors vs observations for the final population pharmacokinetic model of meropenem: (**a**) plots for all subjects (n = 26), (**b**) plots excluding the two patients with extreme values (n = 24).
